# Entry Properties and Entry Inhibitors of a Human H7N9 Influenza Virus

**DOI:** 10.1371/journal.pone.0107235

**Published:** 2014-09-15

**Authors:** Youhui Si, Jianguo Li, Yuqiang Niu, Xiuying Liu, Lili Ren, Li Guo, Min Cheng, Hongli Zhou, Jianwei Wang, Qi Jin, Wei Yang

**Affiliations:** Ministry of Health Key Laboratory of Systems Biology of Pathogens, Institute of Pathogen Biology, Chinese Academy of Medical Sciences & Peking Union Medical College, Beijing, China; SRI International, United States of America

## Abstract

The recently identified human infections with a novel avian influenza H7N9 virus in China raise important questions regarding possible risk to humans. However, the entry properties and tropism of this H7N9 virus were poorly understood. Moreover, neuraminidase inhibitor resistant H7N9 isolates were recently observed in two patients and correlated with poor clinical outcomes. In this study, we aimed to elucidate the entry properties of H7N9 virus, design and evaluate inhibitors for H7N9 virus entry. We optimized and developed an H7N9-pseudotyped particle system (H7N9pp) that could be neutralized by anti-H7 antibodies and closely mimicked the entry process of the H7N9 virus. Avian, human and mouse-derived cultured cells showed high, moderate and low permissiveness to H7N9pp, respectively. Based on influenza virus membrane fusion mechanisms, a potent anti-H7N9 peptide (P155-185-chol) corresponding to the C-terminal ectodomain of the H7N9 hemagglutinin protein was successfully identified. P155-185-chol demonstrated H7N9pp-specific inhibition of infection with IC_50_ of 0.19 µM. Importantly, P155-185-chol showed significant suppression of A/Anhui/1/2013 H7N9 live virus propagation in MDCK cells and additive effects with NA inhibitors Oseltamivir and Zanamivir. These findings expand our knowledge of the entry properties of the novel H7N9 viruses, and they highlight the potential for developing a new class of inhibitors targeting viral entry for use in the next pandemic.

## Introduction

The emergence of a severe human illness caused by a novel avian influenza H7N9 virus has recently been reported in China [1]. Although H7 viruses have occasionally been found to infect humans (H7N2 [2], H7N3 [3] and H7N7 [4], [5]), no human infections with H7N9 viruses have been reported previously [6]. As of August 12 2013, a total of 135 laboratory-confirmed patients were officially reported in mainland China, and 44 (32.6%) of them had died [7]. A large portion of the infected people had a history of poultry exposure [1], [8], even though H7N9 viruses are considered epidemic and low-pathogenic in poultry. Sequence analyses have shown that H7N9 viruses have several molecular signatures of adaptation to grow in mammalian species, including the ability to bind to mammalian cell receptors and to grow at temperatures close to the normal mammalian body temperature [9]. Moreover, the H7N9 virus contains an internal gene cassette from an H9N2 virus [10], which has the ability to infect and move rapidly between numerous avian and mammalian hosts [11]. Thus far, H7N9 has not been found to be transmissible from human to human but should be closely watched in the future.

The neuraminidase (NA) inhibitors are currently available for the treatment of H7N9 virus infection. However, the antiviral resistant H7N9 isolates with NA R292K mutant were recently observed in two patients and correlated with poor clinical outcome. It is with high possibility that the H7N9 virus will be reemerging in the next flu season. Therefore, discovering novel antiviral targets and drug candidates are urgently anticipated for this high lethal viral disease. The entry of influenza virus into host cells establishes the first step of the whole viral life cycle and represents a promising target for novel antiviral drug development. This study was aimed to elucidate the entry characteristics of H7N9 virus, design and evaluate inhibitors for H7N9 virus entry.

## Materials and Methods

### Cells, plasmids, peptides and reagents

The MDCK, A549, NCI-H1650, 293T, Hela, Vero E6, CHO and NIH3T3 cells were purchased from The American Type Culture Collection (ATCC, Manassas, VA, USA). The Huh7.5.1 cells were provided by Dr Francis V. Chisari (Scripps Research Institute, La Jolla, CA, USA), and the chick embryo fibroblast (CEF) cells were derived from specific-pathogen-free fertilized eggs purchased from Vital River Laboratory Animal Technology Co., Ltd. Fibroblasts were prepared from 12-day old embryos under a specific procedure for the chicken embryo collection approved by the Ethics Committee of the Institute of Pathogen Biology. All the cells were maintained in DMEM medium (Invitrogen, Carlsbad, CA, USA) supplemented with 10% fetal bovine serum (Gibco, Carlsbad, CA, USA), 1% nonessential amino acids (Gibco, Carlsbad, CA, USA), and 1% penicillin and streptomycin (Gibco, Carlsbad, CA, USA) at 37°C under 5% CO_2_.

pCAGGS.MCS-based expression plasmids carrying codon-optimized NA or HA genes for influenza A virus A/Brisbane/59/2007 (H1N1), A/California/04/2009 (H1N1), A/Brisbane/10/2007 (H3N2), A/Anhui/1/2005 (H5N1) and A/Anhui/1/2013 (H7N9) were constructed by inserting synthesized sequences into KpnI/SacI restriction sites of pCAGGS [12] (addgene).

Peptides for P155-185, B7^NP^, GBVA10-9 and Scrambled controls were synthesized by standard Fmoc-solid phase methods at Scilight Biotechnology LLC (Beijing, China). The cholesterol moiety was attached to P155-185 via a reaction between the thiol group of an extra cysteine residue and added C-terminally to P155-185 as well as a bromoacetyl derivative of cholesterol (P155-185-Chol).

Chloroquine diphosphate and Zanamivir hydrate were purchased from Tokyo Chemical Industry Co., Ltd (Tokyo, Japan). Bafilomycin A1 and dynasore hydrate were obtained from Sigma-Aldrich (St Louis, MO, USA). Oseltamivir carboxylate was from Hoffmann-La Roche, Ltd (Basel, Switzerland).

### Production and optimization of HA-mediated pseudovirions (HANApp)

Lentiviral pseudotyped particles were produced in 293T cells (70% confluence) and co-transfected with pNL-4.3-Luc-R^-^E^-^, pCAGGS-HA and pCAGGS-NA constructs using a standard PEI-based transfection protocol [13], [14], [15]. Following transfection, the 293T cells were washed once with phosphate-buffered saline and maintained in Opti-MEM I Reduced Serum Medium (Gibco, Carlsbad, CA, USA). For optimizing the infectivity of the produced pseudotyped particles, viral supernatants were generated in 6-well plates as described above. To determine the optimal transfection quantities of pCAGGS-HA and pCAGGS-NA plasmids, one plasmid amount was maintained at 0.5 µg, while the other was varied at 0.1 µg, 0.25 µg, 0.5 µg, 1 µg or 1.5 µg. The culture supernatants were collected at different times post-transfection (24 h, 36 h, 48 h) and incubated with a series of concentrations (0, 2, 4, 6, 8, 10 µg/ml) of TPCK-treated trypsin (Sigma-Aldrich, St. Louis, MO, USA) for 1 h at 37°C. The final culture supernatants were filtered through a 0.45 µm filter (Millipore, Billerica, MA), and aliquots were stored at -70°C until use.

### Pseudotyped particle neutralization assay

H7N9pp was mixed with serially diluted neutralized monoclonal antibodies against H1, H3, H5 or H7, which was provided by Dr Li Guo, [12], [16] for 1 h at 37°C followed by incubation with MDCK cells cultured in a 96-well plate (1×10^4^ cells per well) for 3 h before changing the media. Isotype IgG was included as a control. Infectivity was evaluated at 48 h post-infection by a luciferase assay.

### HANApp entry assay

The pseudotyped viral supernatants prepared above were incubated with cells in 96-well plates for 3 h at 37°C before changing the media. Infectivity was evaluated at 48 h post-infection by a luciferase assay (Promega, Madison, WI, USA) and normalized to that of MDCK cells. To test the antiviral activity of the indicated synthetic peptides, HANApp was treated with serially diluted peptides for 0.5 h at 37°C, and then incubated with MDCK cells in 96-well plates for 3 h at 37°C before changing the media. To determine the effects of the chemicals on virus infection, MDCK cells were seeded into 96-well plates and treated with serially diluted chemicals for 1 h at 37°C followed by infection with HANApp. The dynasore treated group was conducted in Opti-MEM I Reduced Serum Medium, while the other groups were in complete growth medium. Infectivity was evaluated at 48 h post-infection by a luciferase assay.

### Superinfection assay

293T cells were transfected in 96-well plates (70% confluence) with various amounts of pCAGGS-N9 (0 µg, 0.0375 µg, 0.075 µg and 0.15 µg) using a standard PEI-based transfection protocol. Twenty-four hours post-transfection, N9-expressing cells were infected with HANApp as described above [13]. Vesicular stomatitis virus G protein-pseudotyped particles (VSVGpp) were included as a control. For the NA inhibitor treatments, 293T cells were transfected in 96-well plates (70% confluence) with 0.15 µg pCAGGS-N9 using PEI. Four hours post-transfection, the cells were washed with pre-warmed PBS, and 100 µl of fresh media containing the indicated concentrations of Zanamivir hydrate were added to each plate followed by infection with H7N9pp as described above. Infectivity was evaluated at 30 h post-infection by a luciferase assay.

### H7N9 influenza virus infection

The A/Anhui/1/2013 (H7N9) viruses were provided by the CDC, China. The viruses were isolated and passaged in 9-day-old specific pathogen-free chicken embryos. All the experiments with H7N9 viruses were performed in approved enhanced biosafety level 3 (BSL-3) containment laboratories. The H7N9 influenza virus was treated with 2 µM P155-185, P155-185-Chol or scrambled peptide for 0.5 h at 37°C, followed by incubation with MDCK cells cultured in 24-well plates (4×10^5^ cells per well) at 0.1 MOI. The inoculum was removed at 2 h post-infection, and the cells were washed twice with DMEM prior to the addition of Opti-MEM I Reduced Serum Medium containing 1 µg/ml TPCK-trypsin with or without 100 nM Zanamivir hydrate (ZA) or 200 nM Oseltamivir carboxylate (OS). Treatment of H7N9 influenza virus with 5 µM chloroquine diphosphate, 2.5 nM bafilomycin A1 or 10 µM dynasore hydrate was also performed as described above. H7N9 influenza virus RNA copies were quantified at 24 h post-infection by qRT-PCR according to the WHO protocol for the detection of Avian Influenza A (H7N9) Virus. The cytopathic effects were visually inspected under an inverted microscope at 48 h post-infection.

### Statistical analyses

The data are expressed as the means ± SEM, and all the experiments were repeated at least three times. The data were analyzed with unpaired two-tailed *t* tests with SPSS 16 and GraphPad Prism 5 software.

## Results

### Production of the H7-pseudotyped particle system

**Figure 1:**
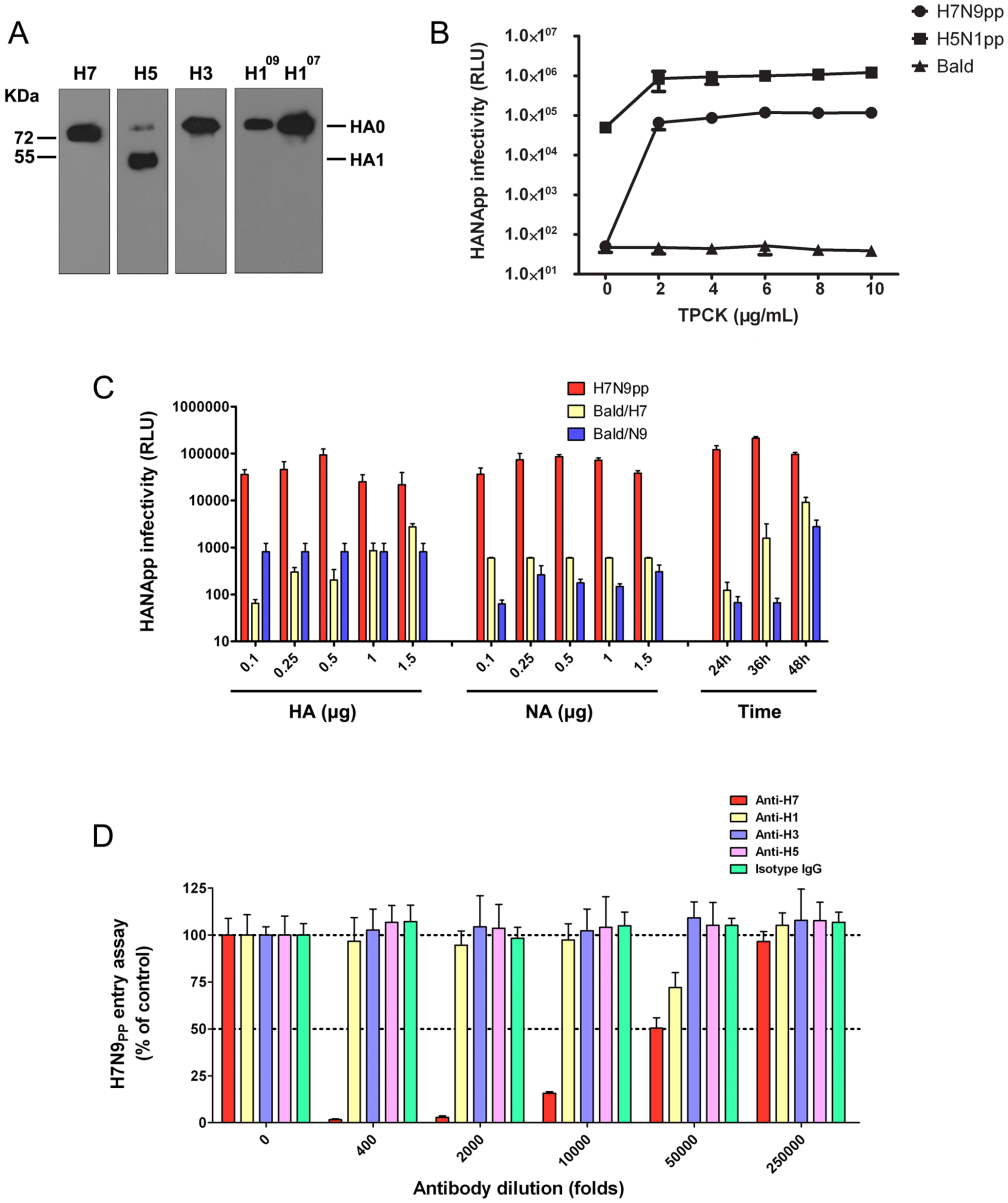
Production and characterization of the H7-pseudotyped particle system. **(A)** Western blot analysis was performed using lysates from 293T cells transfected with the recombinant pCAGGS plasmids expressing H7 (A/Anhui/1/2013), H5 (A/Anhui/1/2005), H3 (A/Brisbane/10/2007), H1^09^ (A/California/04/2009) and H1^07^ (A/Brisbane/59/2007). Specific bands corresponding to HA0 and HA1 are indicated on the right. **(B)** TPCK-treated trypsin treatment is required for H7N9pp infection. The infectivities of H7N9pp and H5N1pp were determined after treatment with different concentrations of TPCK-treated trypsin (0, 2, 4, 6, 8, 10 µg/ml). Bald virus refers to culture supernatants collected from 293T cells co-transfected with 1.5 µg pNL-4.3-Luc-R^-^E^-^ and 0.5 µg pCAGGS-H7 in the absence of pCAGGS-N9 and was used as a control. **(C)** Optimization of H7N9pp packaging. 293T cells were co-transfected with pNL-4.3-Luc-R^-^E^-^, pCAGGS-HA and pCAGGS-NA constructs. pCAGGS-HA was maintained at 0.5 µg, while pCAGGS-NA was varied at 0.1 µg, 0.25 µg, 0.5 µg, 1 µg or 1.5 µg, and the reverse was also performed. The infectivity of 293T culture supernatants collected from different times (24 h, 36 h, 48 h) post-transfection with 1.5 µg pNL-4.3-Luc-R^-^E^-^, 0.5 µg pCAGGS-HA and 0.5 µg pCAGGS-NA constructs was determined as described above. Bald/H7 refers to culture supernatants collected from 293T cells co-transfected with 1.5 µg pNL-4.3-Luc-R^-^E^-^ and the indicated amount of pCAGGS-HA (In ‘NA(ug)’, Bald/H7 refers to culture supernatants collected from 293T cells co-transfected with 1.5 µg pNL-4.3-Luc-R-E- and 0.5 µg pCAGGS-HA). Bald/N9 refers to culture supernatants collected from 293T cells co-transfected with 1.5 µg pNL-4.3-Luc-R^-^E^-^ and the indicated amount of pCAGGS-NA (In ‘HA(ug)’, Bald/N9 refers to culture supernatants collected from 293T cells co-transfected with 1.5 µg pNL-4.3-Luc-R-E- and 0.5 µg pCAGGS-NA). Both Bald/H7 and Bald/N9 were included as negative controls. **(D)** Antibody neutralization assays. H7N9pp was incubated with serially diluted monoclonal antibodies against H1, H3, H5 or H7 for 1 h at 37°C before inoculation onto MDCK cells. The cells were lysed, and luciferase activity was determined at 48 h post-infection. Viral entry in the absence of antibody (0 Ab) was set at 100%. Isotype IgG was included as a negative control.

Hemagglutinin (HA) sequence alignments of influenza A virus A/Brisbane/59/2007 (H1N1), A/California/04/2009 (H1N1), A/Brisbane/10/2007 (H3N2), A/Anhui/1/2005 (H5N1) and A/Anhui/1/2013 (H7N9) were analyzed. The HA cleavage site sequence of H7N9 is P-K-G-R, which is consistent with low pathogenic avian influenza H7 [17]. Western blot analysis also showed that, in contrast to H5N1, the H7N9 HA0 protein cannot be effectively cleaved into functional HA1 and HA2 subunits by intracellular proteases (Fig. 1A). Because HA0 cleavage is a prerequisite for viral entry, we treated the HA pseudotyped particles (HANApp) with a series of concentrations of TPCK trypsin to improve the infectivity of HANApp. As shown in Fig. 1B, in the absence of TPCK-treated trypsin, the H7N9pp was completely non-infectious. However, after incubation with 2 µg/ml TPCK-treated trypsin, H7N9pp could efficiently infect the cells, whereas the proteolysis mode and entry of H5N1pp was quite different from the other tested viruses.

To further optimize the H7N9pp system, we systematically tested the effects of various HA/NA ratios and virus harvest times on the infectivity of H7N9pp. As shown in Fig. 1C, the 293T culture supernatants collected from 24 h post-transfection with 1.5 µg pNL-4.3-Luc-R-E-, 0.5 µg pCAGGS-HA and 0.5 µg pCAGGS-NA constructs exhibits high infectivity with lowest background. To determine whether the produced H7N9pp could mimic the influenza virus entry process, an antibody-mediated neutralization assay was performed. As shown in Fig. 1D, H7N9pp entry was specifically inhibited by an antibody against H7 but not antibodies against H1, H3 or H5 [12]. These findings suggest that H7N9pp-mediated infection has similar properties to H7N9 and thus can be used as an alternative research model for the early events of H7N9 infection.

### Hemagglutinin-mediated entry tropism of H7N9

**Figure 2:**
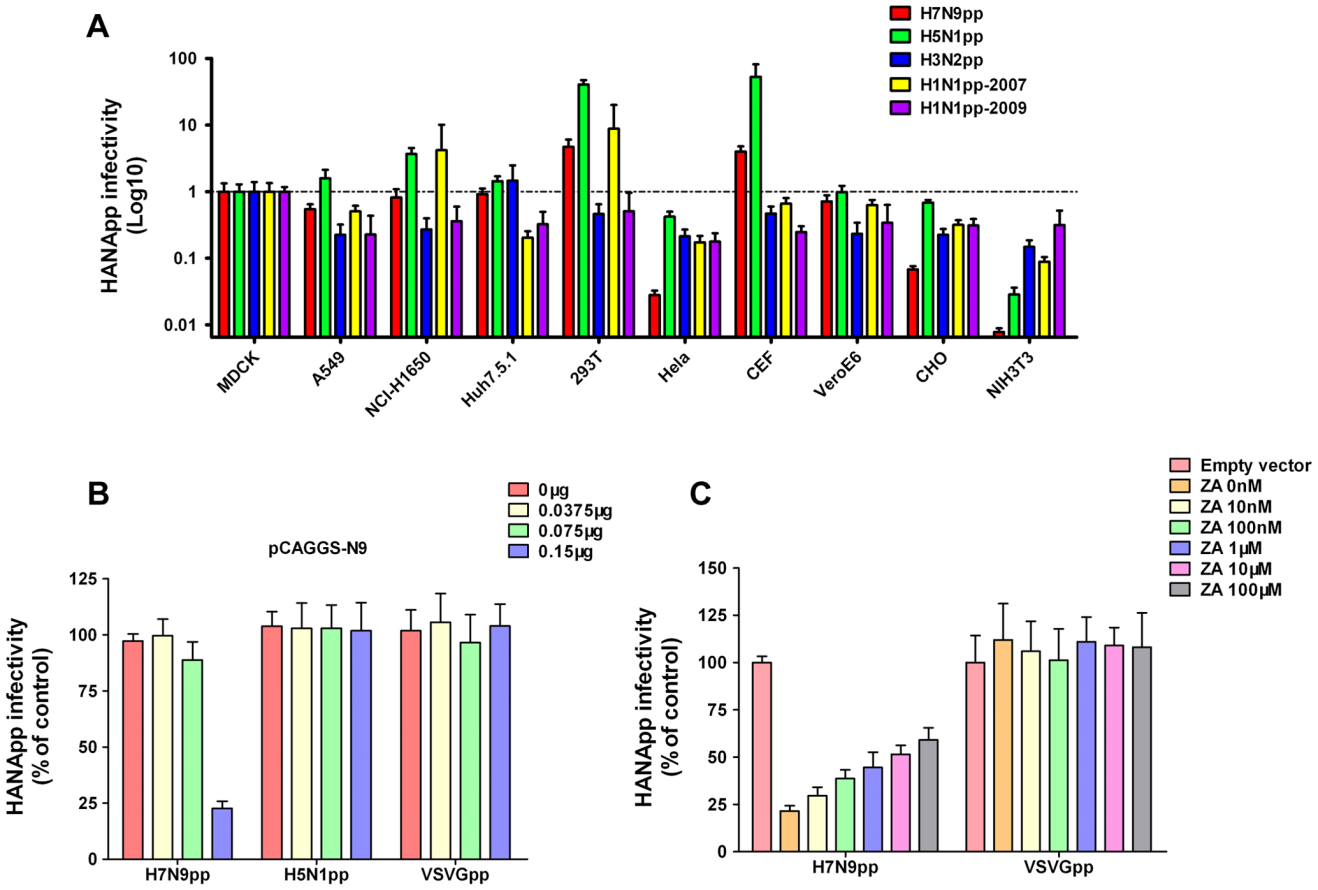
Cell permissiveness to H7N9pp and N9-mediated H7N9pp superinfection exclusion. **(A)** Different subtypes of HANApp with equal amounts of p24 antigen were incubated with various cells derived from different species and tissues, and the transduction efficiencies were measured and normalized to that of MDCK cells. **(B)** N9 expression specifically inhibits H7N9pp infection. 293T cells were transfected with various amounts of pCAGGS-N9 (0 µg, 0.0375 µg, 0.075 µg and 0.15 µg) and then infected with equal amounts of H7N9pp at 24 h post-transfection. Pseudovirus infection was determined by a luciferase activity assay and normalized to that of cells transfected with vector alone. VSVGpp was included as negative control. **(C)** N9-mediated H7N9pp inhibition can be partially blocked with NA inhibitor Zanamivir (ZA). N9-expressing 293T cells were treated with the indicated concentrations of ZA and then infected with H7N9pp. Pseudovirus infection was determined by a luciferase activity assay and normalized to that of cells transfected with vector alone. VSVGpp was included as a negative control.

Recent studies have demonstrated that the novel H7N9 virus can bind to both avian (α2,3-linked sialic acid) and human (α2,6-linked sialic acid) receptors [18], [19]. It remained unknown whether the unique characteristics of H7N9 receptor binding could affect host tropism with regards to viral entry. Therefore, the entry efficiencies of various HANApp were compared using multiple species and tissue-derived cells (Fig. 2A). The infection efficiencies of each H7N9pp were measured and normalized to that of MDCK cells. Among the 10 examined cell types, chicken embryonic fibroblasts (CEF) and human 293T cells were highly susceptible to H7N9pp. Cells derived from human lung (A549 and NCI-H1650), human hepatoma (Huh7.5.1), African green monkey kidney (Vero E6) and Madin-Darby canine kidney (MDCK) supported moderate H7N9pp infections. However, cells of mouse kidney (NIH3T3), hamster ovary (CHO) and human cervical carcinoma (Hela) were nearly non-permissive to H7-mediated infection.

### Neuraminidase N9 limits H7-mediated infection

It has been reported that NA is essential for virus release from host cells [20], and the expression of NA alone can inhibit influenza virus superinfection by removing the SA receptor from the cell surface [13]. To investigate whether H7N9 neuraminidase N9 could inhibit H7N9pp infection, 293T cells expressing various amounts of N9 were infected with H7N9pp, and infectivity was evaluated at 30 h post-infection (Fig. 2B). H7N9pp entry was specifically inhibited in a N9 dosage-dependent manner, whereas the entry of VSVGpp and H5N1pp were unaffected. This N9-mediated H7N9pp superinfection exclusion was partially blocked by the treatment of Zanamivir, a NA inhibitor (Fig. 2C). These results suggest that N9 neuraminidase may play an important role in regulating H7N9 superinfection, and wild type N9 is sensitive to NA inhibitors.

### Inhibition of H7N9 virus by peptides and chemicals

**Figure 3:**
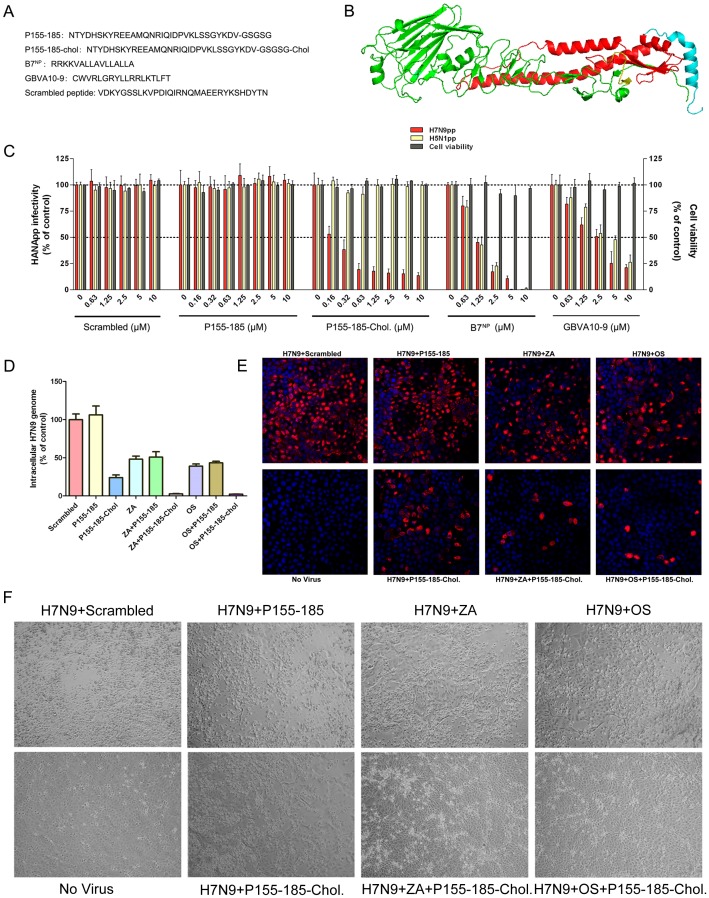
Inhibition of H7N9 virus by peptides. **(A)** Amino acid sequences of the indicated synthetic peptides. **(B)** Location of P155-185. Structure of HA. HA1, green; HA2, red; fusion peptide, yellow; P155-185, cyan. **(C)** Characterization of the antiviral activity of the synthetic peptides. Viral entry without peptide treatment was set at 100%. A scrambled peptide was included as a negative control. Cytotoxicity of the peptides to MDCK cells was measured by a MTT assay. **(D)** The inhibitory effect of P155-185-chol against H7N9 influenza virus in combination with neuraminidase inhibitors. P155-185-chol 2 µM; P155-185 2 µM; scrambled peptide 2 µM; ZA, 100 nM Zanamivir hydrate; OS, 200 nM Oseltamivir carboxylate. **(E)** The same experiments as panel D were performed. H7N9-infected MDCK cells were fixed at 36 h after the indicated treatments and processed for immunostaining and observed under confocal microscope. Fixed cell monolayers were immunostained with an anti-influenza A nucleoprotein (NP) murine monoclonal antibody (MAB8251; Millipore) and Alexa 594 conjugated secondary antibody (red). Nuclei were stained with DAPI (blue). **(F)** The same experiments as panel D were performed. The cytopathic effect of H7N9-infected MDCK cells in the presence or absence of the indicated treatments was visually inspected under an inverted microscope at 48 h post-infection.

To screen for drug candidates against this novel H7N9 virus, we assessed the antiviral potential of several peptides and chemicals using the H7N9pp system *in vitro*. The amino acid sequences of related synthetic peptides are listed in Fig. 3A. As shown in Fig. 3C, P155-185-chol corresponding to the C-terminal ectodomain of the H7 protein (Fig. 3B) showed significant inhibitory effects against H7N9pp infection but not H5N1pp infection, suggesting that the antiviral activity of P155-185-chol was specific to H7. The IC_50_ value of P155-185-chol to H7N9pp was approximately 0.19 µM. However, the unconjugated P155-185 peptide had no inhibitory effects on either H7N9pp or H5N1pp infection. In addition, we tested several previously reported antiviral peptides for H7N9pp inhibition. B7^NP^, a peptide derived from the fibroblast growth factor 4 signal sequence [21], [22], exhibits broad-spectrum antiviral activity against influenza viruses *in vitro*. It was demonstrated that the peptide specifically binds to the viral hemagglutinin protein to inhibit the attachment to the cellular receptor, preventing infection. GBVA10-9, a peptide derived from GB virus A [23], may influence virus entry by affecting the integrity of host cell and virus membranes. It has been reported that the effect of GBVA10-9 on HCV entry is genotype independent. Both peptides also showed inhibitory effects against both H7N9pp and H5N1pp infections in a concentration-dependent manner at low micromolar levels (Fig. 3C).

To further investigate whether P155-185-chol was effective against live H7N9 virus propagation *in vitro* when treated alone or in combination with well-characterized NA inhibitors, the A/Anhui/1/2013 (H7N9) virus infection model in MDCK cells was used. Intracellular viral RNA copies, expression of influenza nucleoprotein (NP) and cytopathic effects were measured. As shown in Fig. 3D, 3E and 3F, A/Anhui/1/2013 (H7N9) virus replication in MDCK cells was effectively inhibited by single drug treatments of P155-185-chol (2 µM), ZA (100 nM) and OS (200 nM), respectively. Surprisingly, when P155-185-chol was used in combination with ZA or OS, A/Anhui/1/2013 (H7N9) virus replication was nearly completely blocked (3D, 3E and 3F).

**Figure 4:**
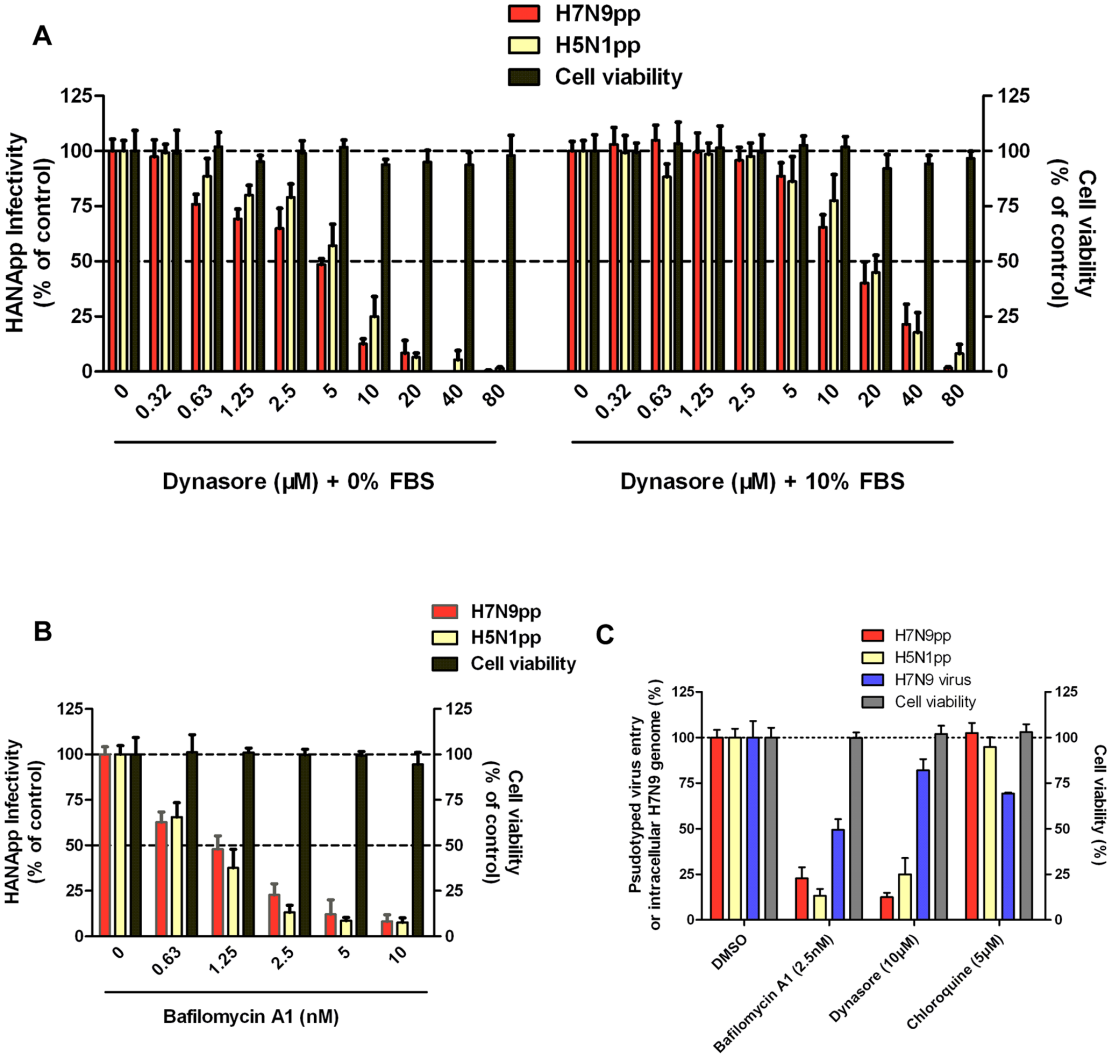
Inhibition of H7N9 virus by chemicals. **(A)** Inhibition of HA-mediated infection by dynasore. Dynasore's concentrations range from 0 to 80 µM in the absence (left) or presence (right) of 10% fetal bovine serum (FBS). MDCK cells were kept at 37°C in Opti-MEM I Reduced Serum Medium with or without 10% FBS at all times. Dynasore was added 1 h prior to HANApp infection. Pseudovirus infection was determined by a luciferase activity assay 48 h post-infection. Viral entry in the 0 µM treatment control group was set at 100%. **(B)** The inhibitory effect of bafilomycin A1. MDCK cells were pretreated with bafilomycin A1 (concentration range 0 nM to 10 nM) for 1 h at 37°C followed by infection with HANApp. Pseudovirus infection was determined by a luciferase activity assay at 48 h post-infection. Viral entry in the 0 nM treatment control group was set at 100%. **(C)** Antiviral effects of bafilomycin A1, dynasore and chloroquine. Viral entry in the DMSO treated control group was set at 100%. The cytotoxicity of the chemicals at indicated concentrations for MDCK cells was also measured by a MTT assay.

A number of cellular events are involved in influenza virus entry. Thus, we next determined whether H7-mediated infection was sensitive to several specific inhibitors. Dynasore [24] (a specific inhibitor of dynamin), bafilomycin A1[25], [26] (a highly specific inhibitor of vacuolar type H+-ATPase) and chloroquine [27], [28] (a 9-aminoquinolone with well-known anti-malarial effects) have been previously reported to inhibit the growth of influenza virus in cell culture. As shown in Fig. 4A, dynasore also showed inhibitory effects on the entry of H7N9pp into MDCK cells. The IC_50_ value of dynasore was 3.84 µM in the absence of fetal bovine serum or 16.89 µM in the presence of 10% fetal bovine serum. Bafilomycin A1 was a powerful inhibitor of H7N9pp infection at 2.5 nM. Concentrations of bafilomycin A1 as low as 10 nM almost completely prevented H7N9pp infection in MDCK cells (Fig. 4B). Similarly, live H7N9 virus exhibited a corresponding reduction upon bafilomycin A1 treatment under the same condition. However, the inhibitory effects of 10 µM dynasore on H7N9 virus were not significant (Fig. 4C). Chloroquine had no effects on H7N9pp entry but conferred a weak inhibition against H7N9 virus at 5 µM. Cytotoxicity assays demonstrated that all of the indicated concentrations of peptides and chemicals did not produce cytotoxicity in MDCK cells (Fig. 4A, 4B and 3C).

## Discussion

The human infection with H7 subtypes of influenza viruses mainly resulted in conjunctivitis and mild upper respiratory symptoms. However, the recently H7N9 outbreak in China caused high lethal rate. HA is synthesized as a precursor HA0, which is subsequently cleaved into HA1 and HA2 for its full function [29], [30]. It has been demonstrated that the cellular proteolytic conversion of HA0 to HA1 and HA2 is an essential step for viral entry and multiplication within the infected host and thus is associated with pathogenicity of influenza viruses [31], [32]. In the present study, H7N9 possess an HA cleavage site with a monobasic motif susceptible to only several trypsin-like proteases limited in a few cell types. These suggested that the existence of a multi-basic cleavage site is not essential for the high pathogenicity of avian influenza virus in humans.

To facilitate H7N9 study, we developed an H7N9-pseudotyped particle system bearing virus HA and NA glycoproteins. The produced H7N9pp was neutralized specifically by an antibody against H7 but not antibodies against H1, H3 or H5. In addition, H7N9pp infection was also sensitive to bafilomycin A1 and dynasore as well as other influenza A viruses. These findings suggest that H7N9pp could mimic the influenza virus entry process. Recent studies have demonstrated that the novel H7N9 virus can bind to both avian-type (α2,3-linked SA) and human-type (α2,6-linked SA) receptors. These presented us questions about whether the changed Receptor-Binding ability of the novel H7N9 viruses can affect tropism and pathogenesis of these viruses in mammals. However, the HA-mediated entry assay indicated that the tropism of H7N9 was quite similar to avian H5N1 rather than human pandemic influenza. Until now, information is still limited about the transmission and pathogenicity associated with this novel avian influenza H7N9 virus.

Currently, the NA inhibitors Oseltamivir and Zanamivir are the front-line therapeutic options against this novel H7N9 influenza virus, which contains an S31N mutation in its M2 protein and thus confers resistance to the M2 ion channel blockers [1]. However, recent studies have reported that H7N9 isolates with the R292K mutation are resistant to Zanamivir, Peramivir and Oseltamivir [33]. Two of 14 patients infected with the novel H7N9 influenza virus possessing an R292K NA mutation had a poor clinical outcome [34]. Therefore, discovering novel antiviral targets and drug candidates is urgently expected. HA-mediated virus-host membrane fusion is a prerequisite for the viral life cycle and is a promising antiviral target. Based on the HA-dependent membrane fusion model [35], [36], we designed a cholesterol-conjugated peptide, named P155-185-chol, which is derived from the amino acid 155-185 region of H7N9 HA2. P155-185-chol demonstrated H7N9pp-specific inhibition of infection and exhibited significant inhibitory effects against H7N9 virus.

In summary, our study demonstrated that the established H7N9-pseudotyped particle system can largely mimic the entry properties of the H7N9 virus, thus providing a relatively safe experimental system for studying the entry of H7N9 virus and for high-throughput screening of entry inhibitors, such as small molecules, peptides or antibodies. A potent anti-H7N9 peptide (P155-185-chol) was successfully identified in this study. When used in combination with neuraminidase inhibitors (Oseltamivir, Zanamivir), P155-185-chol showed additive and significant inhibitory effects against H7N9. By virtue of its distinct mechanisms of inhibition, P155-185-chol may be further optimized to improve its activity, highlighting a potential therapeutic candidate for H7N9 infections that can be used in combination with other anti-flu drugs.
